# Cortical maturation from childhood to adolescence is reflected in resting state EEG signal complexity

**DOI:** 10.1016/j.dcn.2021.100945

**Published:** 2021-03-23

**Authors:** Stefon van Noordt, Teena Willoughby

**Affiliations:** a Azrieli Centre for Autism Research, Montreal Neurological Institute and Hospital, McGill University, Montréal, Canada; b Department of Psychology, Brock University, St. Catharines, Ontario, Canada

**Keywords:** Maturation, EEG, Multiscale entropy, Development

## Abstract

Endogenous cortical fluctuations captured by electroencephalograms (EEGs) reflect activity in large-scale brain networks that exhibit dynamic patterns over multiple time scales. Developmental changes in the coordination and integration of brain function leads to greater complexity in population level neural dynamics. In this study we examined multiscale entropy, a measure of signal complexity, in resting-state EEGs in a large (N = 405) cross-sectional sample of children and adolescents (9–16 years). Our findings showed consistent age-dependent increases in EEG complexity that are distributed across multiple temporal scales and spatial regions. Developmental changes were most robust as the age gap between groups increased, particularly between late childhood and adolescence, and were most prominent over fronto-central scalp regions. These results suggest that the transition from late childhood to adolescence is characterized by age-dependent changes in the underlying complexity of endogenous brain networks.

## 1 Introduction

The transition to adolescence is marked by substantial maturational changes in brain structure and function. Developmental neuroimaging studies reveal that grey matter volume peaks following the transition into adolescence and decreases with subsequent aging (Blakemore, 2012a, b; Lebel and Beaulieu, 2011). The reductions in grey matter are coupled with increases in white matter volume, structural integrity, and brain network organization (Blakemore, 2012b; Váša et al., 2018; Wilke et al., 2007). As part of a complex and dynamic system these structural changes are regionally specific and not always linear (Lebel and Beaulieu, 2011), but generally show an ontogenetic topographical organization with early changes in posterior regions followed by later changes in anterior regions (Gogtay et al., 2004; Shaw et al., 2008). As development progresses, the widespread pruning, myelination of long-range networks, and changes in synaptic connectivity (Luna and Sweeney, 2004; Stevens, 2009; Váša et al., 2018) result in refined segregation and integration of information processing in the brain.

In many ways, neural development is seen as an emergent property where localized processes become more integrated and distributed across functional brain networks (Smith and Thelen, 2003). The cortex is highly modular such that computation in localized regional circuity is integrated with distributed networks across multiple spatial and temporal scales (Muldoon et al., 2016; Sporns, 2011). These changes in neural architecture provide a balance between local differentiation and global integration (Sporns, 2011) that is required for increasing one’s proficiency in adaptive responding to complex and changing environments. Increased coordination between localized and distributed processing is thus a property of neural development that subserves learning (Deco et al., 2013) and allows for adaptation to environmental uncertainty (Grady and Garrett, 2018).

The increased precision in coordination and integration of brain function across development leads to increased variability in population level neural dynamics (McDonough and Nashiro, 2014; Misic et al., 2011; Vakorin et al., 2011). Variability in many physiological processes is thought to reflect the capacity for the system to respond to environmental demands, leading to a level of complexity that is required for distributed processing and integration (Golos et al., 2015; McIntosh et al., 2010). Normative developmental changes in brain structure and function support increased information processing capacity, represented by shifts from stability to a more variable and stochastic state. This dynamic property of neural activity is thought to result from functional network re-configurations and is reflected in endogenous brain states (McIntosh et al., 2010).

A growing body of work has focused on brain signal complexity as a marker of maturation. Multiscale entropy (MSE), which measures the amount of signal similarity across a time series, was introduced for the analysis of physiological signals that are typically non-stationary and has shown promise in capturing the complexity of multiple biological processes (Costa et al., 2002, 2005). MSE is a measure of signal complexity that considers the degree to which the signal is predictable over progressively longer temporal scales, assigning low values to fully periodic as well as and random fluctuations. For example, white noise has extremely low complexity as the highly random fluctuations do not contain regular patterns in their dynamics over time. Biological signals often contain non-linear activity that has a structure, or patterns of structures, that span across frequencies and temporal scales. Several development studies have shown that complexity is a marker of functional brain network organization (McIntosh et al., 2010; McIntosh et al., 2008), increasing from early infancy into childhood with different maturational trajectories across sensory modalities (Lippe et al., 2009). Other studies have shown that increased variability in functional complexity maps on to maturation of stable behavioural responses (McIntosh et al., 2008; Mišić et al., 2010). In perhaps the largest cross-sectional sample of children to date (*N* = 464, ranging from 7 to 11 years of age), Miskovic et al. (2015, 2016) also found age-dependent increases in the density, spatial distribution, and signal complexity of endogenous functional brain networks. Taken together, these studies suggest that neural signal complexity tracks developmental changes in functional brain networks that mediate behaviour.

Despite these important findings in childhood, studies have yet to examine whether the well-known changes in brain networks from childhood to adolescence, including the extensive strengthening and reorganization of neural circuitry across networks leading to greater flexibility, efficiency and specialization (Casey et al., 2019; Ernst et al., 2015), are reflected in the dynamics of signal complexity. Here we aimed to extend previous work on normal human brain development by using a large cross-sectional cohort to examine resting state brain signal variability from late childhood into adolescence. We expected that brain signal variability would increase with age and extend from fine to coarse time scales. We expected that these changes would be most prominent in anterior compared to posterior regions, reflecting the maturation of higher-order association areas from childhood to adolescence.

## 2 Methods

### 2.1 Participants

Participants included children and adolescents aged 8–16, who were recruited through elementary and secondary schools as part of a larger study called the Brock Healthy Youth Project (BHYP). Parents were asked to identify whether their child had any illnesses or disabilities (either physical or mental). Two participants were excluded because of a diagnosis of autism, one participant was excluded because they are prone to seizures, and one participant was excluded because of a diagnosis of cerebral palsy. The sample for the current study included 467 participants representing the first wave of data collection. Some participants were excluded from the analyses due to excessive artifact identified during pre-processing, not having a minimum of 20 s of continuous artifact free data, or errors in MSE calculation. Four participants who were aged 8 years were also removed as this sample size was deemed too small for adequate hypothesis testing. The final sample resulted in 405 participants. Demographics for the sample are summarized in Table 1.

**Table 1:** Mean (%) of participant demographics by age.

	Age 9	Age 10	Age 11	Age 12	Age 13	Age 14	Age 15	Age 16
	34 (8.4)	73 (18)	72 (17.8)	57 (14.1)	57 (14.1)	62 (15.3)	36 (8.9)	14 (3.5)
***Biological Sex***								
Female	16 (47)	33 (45)	31 (43)	29 (51)	33 (58)	26 (42)	15 (42)	9 (64)
Male	18 (53)	40 (55)	41 (57)	28 (49)	24 (42)	36 (58)	21 (58)	5 (36)
***Race***								
Caucasian	26 (76.5)	62 (85)	55 (76)	43 (75)	44 (77)	38 (61)	15 (41.6)	8 (57)
Black	–	2 (2.7)	3 (4.1)	2 (3.5)	–	1 (1.6)	–	–
Asian	–	–	2 (2.7)	–	–	1 (1.6)	1 (2.7)	–
Hispanic	–	1 (1.4)	1 (1.4)	–	2 (1.2)	2 (3.2)	2 (5.5)	–
Indigenous	–	1 (1.4)	–	1 (1.25)	–	–	–	1 (7)
Mixed	2 (5.8)	4 (5.5)	2 (2.7)	4 (7)	4 (7)	9 (14.5)	3 (8.3)	1 (7)
Prefer not to answer	–	–	–	1 (1.25)	1 (1.2)	1 (1.6)	–	–
***Parental education***								
Some high school	–	–	1 (1.4)	–	–	–	–	–
High school diploma	–	4 (5.4)	1 (1.4)	2 (3.5)	1 (1.7)	2 (3.2)	3 (8.3)	–
Some university/college	4 (11.8)	9 (12.3)	9 (12.5)	6 (10.5)	5 (8.8)	2 (3.2)	3 (8.3)	3 (21.4)
Associate degree/diploma	13 (38.2)	31 (42.5)	32 (44.4)	20 (35)	29 (50.9)	25 (40.3)	8 (22.2)	4 (28.6)
Undergraduate degree	9 (26.4)	19 (26)	21 (29.2)	22 (38.6)	15 (26.3)	26 (42)	17 (5.6)	3 (21.4
Graduate degree	6 (17.6)	10 (7.3)	4 (5.6)	5 (8.8)	4 (7)	4 (6.4)	2 (5.6)	3 (21.4)

Note: Numbers and percentages reflect proportion of cases within a given age category and do not always sum to 100 due to missing response on some variables.

### 2.2 Procedure

Students were invited to participate in the study through research visits to schools. Some of the participants from the larger BHYP study were invited to complete a Mobile Lab component, which involved performing a series of computerized tasks while EEG was recorded. Prior to the experimental tasks, resting state EEG was recorded during one-minute blocks alternating between eyes open and eyes closed. Each block was completed twice, resulting in four minutes of resting state EEG (2 min eyes open, 2 min eyes closed). This study was approved by the University Ethics Board. Participants provided informed assent and parents provided informed consent.

### 2.3 EEG acquisition and data reduction

EEG was continuously recorded using a BioSemi ActiveTwo system with 96 channel dense array montage and 7 exogenous sensors placed on the face. Automated pre-processing was done using the Lossless Pipeline (https://github.com/BUCANL/BIDS-Lossless-EEG), implemented in EEGLAB and executed in Octave on Compute Canada’s national computing cluster Cedar. Pre-processing involved comprehensive data annotation to identify artifacts and non-stationarity in scalp channels and independent components. Quality control involved expert review of all data annotations and confirmation of flagged components informed by ICLabel classification, topographies, continuous time course activation, dipole fit, and power spectrum. For an expanded description of pre-processing criteria and artifact thresholds, see (Heffer and Willoughby, 2020).

Once flagged ICs were removed from the data, the re-constituted scalp EEG was low pass filtered at 30 Hz and segmented into 20 s non-overlapping windows (20,480 data points with sampling rate of 512 Hz). Each of these 20 s windows were assessed and rejected based on the presence of extreme channel voltages (+/− 150 mV; *eegthresh* EEGLAB function) and joint probability of channel voltages (5 standard deviations; *jointprob* EEGLAB function). The pre-processing resulted in an average of 1.13 min (SD = 0.02) of data (i.e., 3.4 continuous 20 s epochs) across participants. MSE was then calculated for all channels on each 20 s segment and averaged across segments to produce individual subject MSE estimates.

### 2.4 Multiscale entropy extraction

MSE calculation followed the original implementation from (Costa et al., 2005), which involves calculating sample entropy (Richman and Moorman, 2000) at multiple time scales of the EEG signal. Initially a coarse graining procedure for a given time scale (τ) is performed by calculating the average of neighbouring points from the original time series across non-overlapping windows of length τ. Sample entropy is then quantified at each of the temporal scale factors. Similar to the original implementation and other work, we included scale factors from 1 to 20. As a measure of unpredictability across the duration of a time series, at a given scale factor, sample entropy represents the conditional probability that any two consecutive data sequences of pattern length (*m* + 1) will be the same given a match for the first *m* points. Patterns are deemed to match if the absolute amplitude difference falls in the defined tolerance range, *r*.

Sample entropy is calculated as:

$$S_{E}\left(m,r,N\right)=-\text{In}\left(\frac{c_{m}+1\left(r\right)}{c_{m}\left(r\right)}\right)$$

where:

$$c_{m}\left(r\right)=\frac{\left\{number\;of\;pairs(i,j)\;with\left|x_{i}^{m}-x_{j}^{m}\right|<r,\;\;i\neq j\right\}}{\left\{number\;of\;probable\;pairs=\frac{N-m+1}{N-m}\right\}}$$

Thus, MSE measures the regularity (i.e., unpredictability) of the signal over multiple time scales, such that low values reflect high self-similarity/low complexity and high values reflect irregularity/high complexity. Based on previous developmental research in EEG and MEG studies (Heisz et al., 2012; McIntosh et al., 2008; Mišić et al., 2010; Misic et al., 2011; Miskovic et al., 2016), we set *m* = 2 and *r* = .5.

### 2.5 PSD and power-law scaling extraction

Given that sample entropy estimates are sensitive to linear (i.e., changes in PSD) and non-linear dynamics in the EEG, we also examined PSD changes across development and in relation to MSE. Power spectrum density was calculated from the artifact-free resting EEG in order to examine developmental changes in spectral power scaling (i.e., 1/*f*) and whether power dynamics related to MSE. Continuous resting EEG during eye closed were epoched into 2 s windows to which a modified Welch periodogram was applied, with a Hanning tapered window of 1024 data points and 50 % overlap, to estimate PSD between 1 and 30 Hz. The frequency spectra were transformed into log-log space and the power-law scaling exponent, which represents the line of best fit for frequencies from 1−30 Hz, was estimated by robust linear regression.

### 2.6 Phase-shuffled surrogate time series

**Fig. 1:**
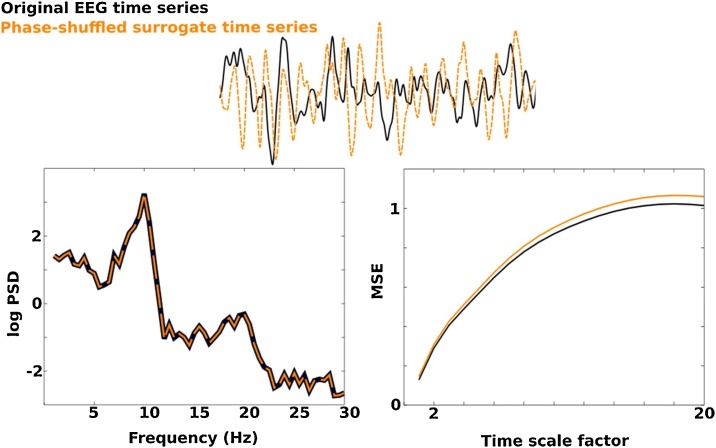
Top panel illustrates a segment of continuous EEG signal (black) and phase-shuffled surrogate time series (yellow) that was generated by the IAAFT algorithm. The power spectrum of these the original EEG and phase-shuffled time series is identical. MSE estimates are sensitive to temporal dependence and yields higher values for the phase-shuffled surrogate time series as it has more irregularity (For interpretation of the references to colour in this figure legend, the reader is referred to the web version of this article.).

To further test whether MSE is sensitive to non-linear temporal dependencies in EEG signal irregularities, we repeated our analysis using phase-shuffled surrogate time series. These surrogate time series were generated by performing an FFT on the original EEG time series, randomly shuffling phase of the Fourier components, and applying the inverse Fourier transform to regenerate the time domain. Given that the power spectrum reflects a linear process, but does not contain phase information, we expect to observe higher entropy when estimated on these randomly phase-shuffled surrogates as the time series has more irregularity than the original EEG time series. We used the iterated amplitude adjusted Fourier transform (IAAFT) algorithm as it has been shown to reduce unreliable identification of nonlinearity (Schreiber and Schmitz, 1996). The phase-shuffled surrogate time series were extracted using a maximum of 100 iterations. See Fig. 1 example comparing original EEG and surrogate time series and Supplementary Fig. 1 for mass univariate results highlighting developmental differences in MSE.

### 2.7 Statistical analyses

To examine global topographical differences MSE values were averaged and submitted to 2000 percentile bootstrap with 20 % trimmed means to test main effects and interactions between Region (frontal, central, parieto-occipital) and Hemisphere (left, right). These analyses were carried out using the *wwmcppb* function from the robust statistics package Hypothesize (Campopiano and Wilcox, 2020).

To test MSE and PSD differences across chronological age, we performed a series of independent samples, two-tailed cluster permutation tests using threshold free cluster enhancement (TFCE; Mensen and Khatami, 2013). TFCE builds on other cluster forming approaches (e.g., cluster max, cluster mass; Bullmore et al., 1999; Groppe et al., 2011a, b; Maris, 2004; Maris and Oostenveld, 2007) by exploiting the temporal and spatial dependencies in EEG data and being sensitive to strong(er)-narrow and weak(er)-broad effects. By taking into account signal intensity (height: H) and spatial distribution (extent: E) across many thresholds, TFCE offers less trade-offs between sensitivity and control of Type I error rate, or the requirement for using potentially arbitrary cut-offs when defining cluster thresholds. Combined with permutation tests, TFCE is a useful method to effectively maintain family-wise alpha at 0.05 and control Type I error rates for multiple comparisons across all cephalic channels and entropy time scale factors. Based on the results from Mensen and Khatami (2013), we set E = .66 and H = 2 for TFCE. For hypothesis testing we used a Monte Carlo approach with 2000 random between-participant permutations were used to establish an empirical distribution that approximates the null hypothesis that no group differences exist. Similar approaches have been widely used in functional MRI analyses to account for the intensity and distribution of signal across neighbouring voxels (Smith and Nichols, 2009; Wu et al., 2011). These analyses were carried out using the *ept_TFCE* function from TFCE toolbox (https://github.com/Mensen/ept_TFCE-matlab).

To test age differences in PSD slopes we conducted a mixed ANOVA with region as the within subjects factor using the *lmer* function in R. Significant effects were followed by post hoc tests using Bonferroni correction for multiple comparisons. The association between PSD slope and MSE were assessed by robust linear regression using the *robustfit* function in MATLAB.

## 3 Results

### 3.1 Topographical distribution of MSE

The spatial distribution of MSE, averaged across time scales, showed central electrodes had greater MSE than frontal (*p* < .001, [.012, .005]) and posterior (*p* < .001, [.009, .019]) electrodes, and that frontal electrodes had greater MSE than posterior sites [*p* < .01, [.002, .014]). Although there was no evidence of a main effect of hemispheric differences, there was an interaction between region and hemisphere. Specifically, MSE was greater in the right hemisphere compared to left for frontal compared to posterior (*p* < .001, [.009, .003]) and for central compared to posterior (*p* < .01, [.008, .002]). There were no differences in hemispheric MSE between frontal and central regions.

### 3.2 Developmental changes in MSE

**Fig. 2:**
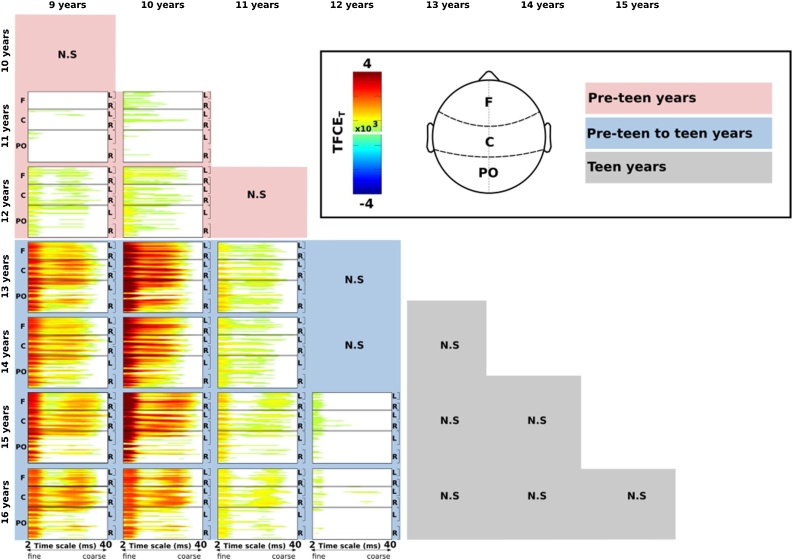
Pairwise age group contrasts for each scalp electrode and time scale factor. Hot colors indicate greater MSE in the older group of a given contrast (e.g., 9 vs 13), masked by significance using Threshold Free Cluster Enhancement with 2000 random between-subjects permutations. No robust effects were observed for decreases in MSE with age. Shaded backgrounds for age group contrasts within pre-teen years (pink), between pre-teen and teen years (blue), and within teen years (grey). Note: F – frontal; C – central; PO – parieto-occipital. L – left hemisphere; R – right hemisphere. N.S. – no significant contrasts (For interpretation of the references to colour in this figure legend, the reader is referred to the web version of this article.).

Differences in MSE in relation to chronological age were tested using a series of mass univariate tests for each electrode and time scale with TFCE (see Fig. 2). As can be seen from the contrast plots, there are consistent increases in brain signal complexity from 9–16 years.

**Fig. 3:**
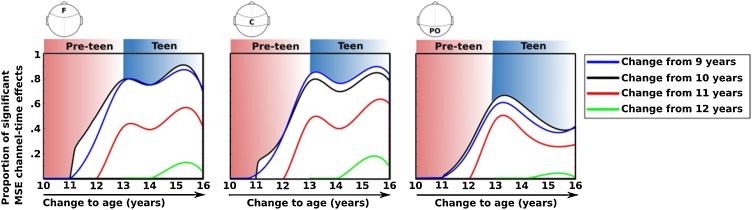
Curves show the proportion of significant increase in MSE from childhood to adolescence across scalp electrodes and time scale factors. Each curve shows the difference from a younger age to an older age, as indicated on the x-axis. The proportion of significant changes are greatest from pre-teen to teen year, particularly for frontal and central channels. Note: F – frontal; C – central; PO – parieto-occipital.

As the age gap between contrasts becomes larger, the differences in MSE increases in magnitude and robustness (i.e., the spatial concentration of effects across channels and time factors), particularly at fronto-central clusters compared to posterior regions. The transition from pre-teen to teen is marked by major changes in EEG complexity, as shown by greater MSE that spans both spatial and temporal scales. A descriptive summary of the developmental change in MSE is shown in Fig. 3, which represents the proportion of channel-by-time scale factor effects that were statistically significant from the TFCE analysis. These plots indicate that robust increases in EEG complexity were most prominent between pre-teen and teen years. Conversely, EEG complexity was relatively stable within childhood and did not show any reliable change within the teen age ranges.

### 3.3 Developmental changes in PSD

**Fig. 4:**
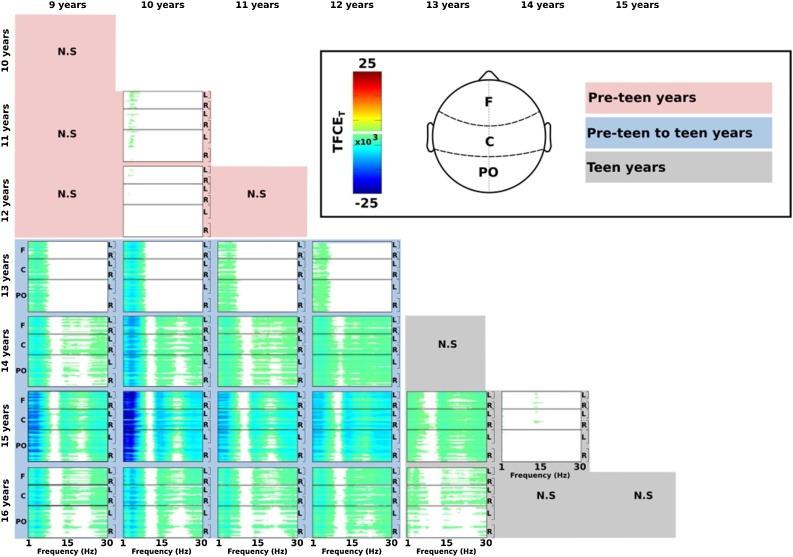
Pairwise age group contrasts for each scalp electrode and frequency. Cold colors (blue) indicate less spectral power (dB) in the older group of a given contrast (e.g., 9 vs 13), masked by significance using Threshold Free Cluster Enhancement with 2000 random between-subjects permutations. Shaded backgrounds for age group contrasts within pre-teen years (pink), between pre-teen and teen years (blue), and within teen years (grey). Note: F – frontal; C – central; PO – parieto-occipital. L – left hemisphere; R – right hemisphere. N.S. – no significant contrasts (For interpretation of the references to colour in this figure legend, the reader is referred to the web version of this article.).

A similar mass univariate approach using TFCE was used to examine differences in frequency spectra in relation to chronological age. Fig. 4 shows age-related changes that indicate a general reduction in spectral power with increasing age. Similar to the MSE results, changes were most prominent in terms of magnitude and channel-frequency span when contrasting pre-teen to teen ages. Reliable changes in spectral power within pre-teen and teen years were relatively sparse or absent. The TFCE results reveal that the reduction in PSD with age is most notable in the delta and theta frequency range (i.e., <10 Hz), with lower magnitude effects in the beta range that emerge in the teen years.

**Fig. 5:**
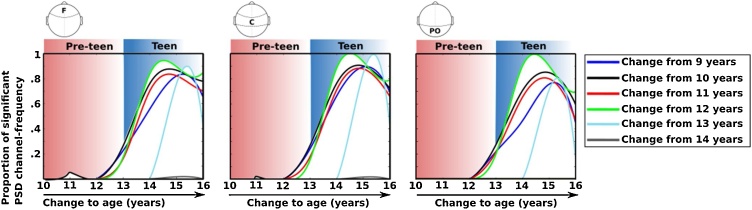
Curves show the proportion of significant decrease in PSD from childhood to adolescence across scalp electrodes and time scale factors. Each curve shows the difference from a younger age to an older age, as indicated on the x-axis. The proportion of significant changes are greatest from pre-teen to teen years. Note: F – frontal; C – central; PO – parieto-occipital.

A descriptive summary of the developmental change in PSD is shown in Fig. 5, which represents the proportion of channel-by-frequency effects that were statistically significant from the TFCE analysis. These plots indicate that robust decreases in PSD were most prominent between pre-teen and teen years.

### 3.4 Developmental changes in PSD slopes

**Fig. 6:**
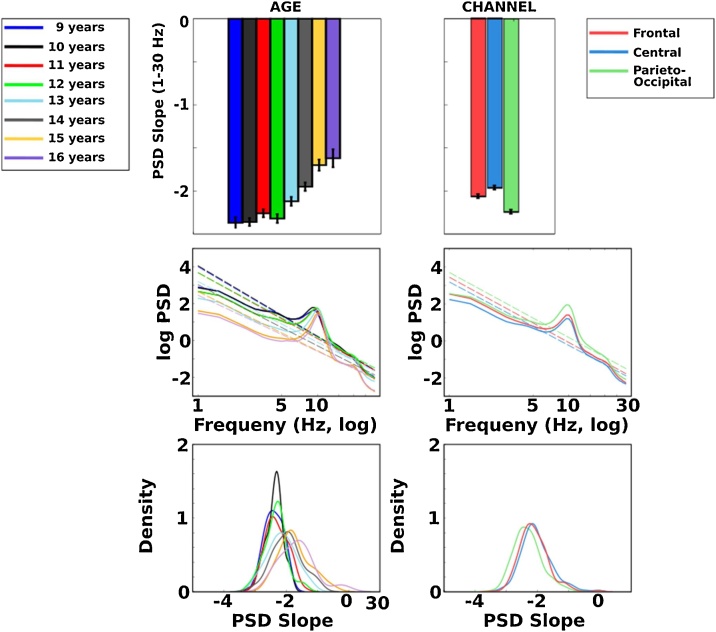
Main effects of age (left panel) and region (right panel) on PSD slopes. Top panel shows mean PSD slope estimates (1-30 Hz), Middle panel shows PSD curves and fitted slopes (dashed lines), Bottom panel shows kernel densities of power-law exponents (PSD slope).

**Fig. 7:**
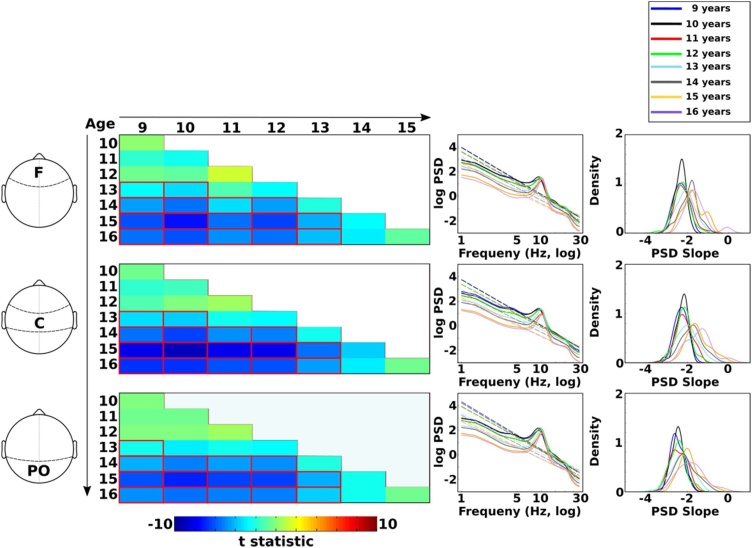
Contrasts for age by regions interactions for PSD slopes. Left panel shows t-statistics for each pairwise age contrast by region. Cold colors (blue) indicate that PSD slope was more negative for the younger age in a given contrast (e.g., 9 vs 10 years). Cells highlighted with red rectangles were statistically significant (Bonferroni corrected for multiple comparisons). Middle panel shows PSD curves fitted slopes (dashed lines). Right panel shows kernel densities of power-law exponents (PSD slope). The pattern of results between chronological ages are the same across all regions, with the exception of the age 10 to 13 contrast which was only significant at frontal and central regions. Note: F – frontal; C – central; PO – parieto-occipital (For interpretation of the references to colour in this figure legend, the reader is referred to the web version of this article.).

A mixed ANOVA revealed significant main effects for region (*F*(7,794) = 213.56, p < .0001, η_p_^2^ = .35 [95 % CI: .31, .39]) and age (*F*(7,397) = 19.64, p < .0001, η_p_^2^ = .26 [95 % CI; .19, .31]). Follow-up tests revealed that PSD slope reliably differed between all regions, with the posterior-occipital cluster showing the most negative slope, followed by frontal and central clusters. Similar to the mass univariate results, the effect of age on PSD slope indicates that pre-teen ages had significantly more negative slopes compared to teens, whereas slope differences within pre-teen and teen ages were less robust or absent. These main effects were superseded by an interaction between age and region (*F*(14,794) = 2.62, p < .001, η_p_^2^ = .04 [95 % CI; .01, .05]). Follow up pairwise comparisons revealed that this interaction was driven by significant differences between 10 and 13 years for frontal and central regions, but not at the parieto-occipital cluster. All other age pairwise comparisons were similar for frontal, central, and posterior-occipital regions. See Fig. 6 for a summary of PSD slope effects for age and region and Fig. 7 for a summary of the age by region interaction effects.

### 3.5 Linking PSD to MSE

**Fig. 8:**
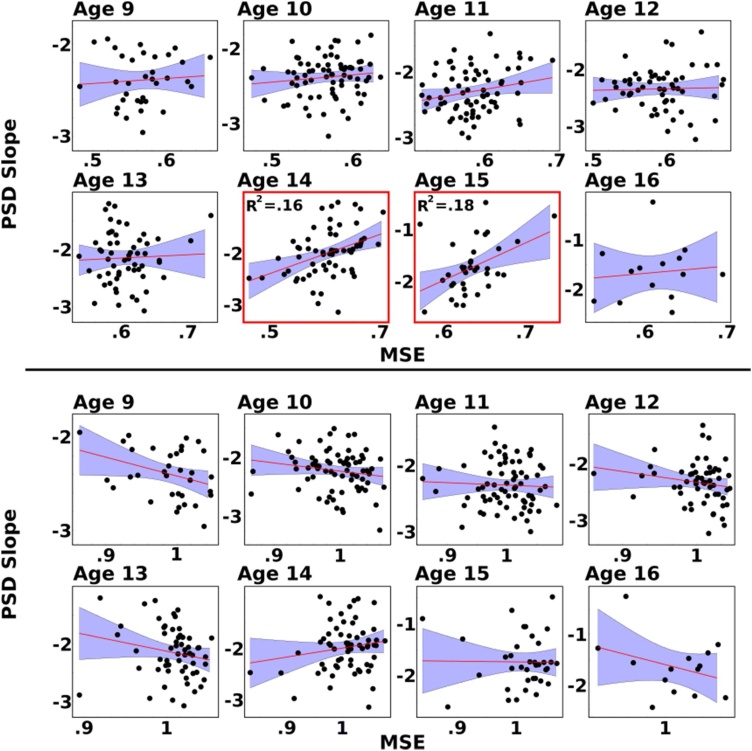
Scatterplots of robust regression of PSD slope predicting MSE at short (top) and long (bottom) time scale factors for each age group. PSD slope explained 16 % and 18 % of MSE estimates over short time scale factors in individuals aged 14 or 15 years, respectively.

Given the developmental and individual differences in PSD, and the partial overlap between PSD and MSE estimates, we examined whether PSD slope was linked to MSE across development. Fig. 8 presents a series of robust regressions to assess the links between PSD slope and MSE at short and long time scale factors. Although we did observe a general trend for most ages whereby PSD slopes were positively related to MSE shorter scales and negatively at MSE longer time scales, PSD slope did not predict MSE estimates with the exception of ages 14 and 15 where PSD slope explained 16 % and 18 % of the variance in MSE short time scale factor, respectively.

## 4 Discussion

We examined normative development of cortical fluctuations in resting state EEG from late childhood to mid-adolescence in a large community sample. Our main findings showed that variability in endogenous cortical dynamics, a proxy for neural complexity, exhibits robust increases across development with the most prominent changes occurring from pre-teen to teen years to adolescence, primarily in fronto-central regions. Significant changes within childhood and within the teenage years were restricted both spatially and temporally, or not significant, suggesting that increases in neural complexity reflect a maturational change in underlying brain networks.

Our mass univariate analyses revealed similar developmental effects for MSE and PSD, such that the differences between pre-teen and teenage years revealed the most consistent and robust effects. EEG signal complexity showed consistent age-related increases, whereas spectral power and PSD slope decreased. Age related changes within the pre-teen and teenage years were reduced in magnitude, relatively sparse in terms of spatial-temporal (MSE) and spatial-frequency (PSD) distribution, or completely absent. The pattern of these results is to be expected given that MSE is sensitive to both linear and non-linear dynamics and changes in PSD partially overlap with entropy estimate. Despite this partial overlap, MSE is differentially sensitive than PSD to irregularities in the EEG and also captures non-linear dynamics that are not captured by spectral power (Miskovic et al., 2015, 2019, Vasily and McIntosh, 2012).

Our findings suggest that developmental changes in signal complexity are sensitive to non-linear dynamics of signal irregularity that are not completely reflected in age related PSD rotations. MSE showed significant increases for from age 9–10 and 11 years, but not for PSD. Conversely, PSD showed widespread attenuation from 13 to 16 years, whereas signal complexity was stable across all scalp regions and temporal scales. Repeating our MSE analysis using randomly phase-shuffled surrogates of the EEG time series resulted in higher entropy estimates and did not eliminate the developmental changes observed in the original EEG time series. Finally, we found that, with the exception of 14 and 15 year olds, individual variation in PSD slope did not consistently predict MSE at either short or long time scales. Together, the current findings suggest that MSE is capable of capturing maturational changes in non-linear temporal dynamics that are not simply due to developmental shifts in PSD. Our results expand previous work on the development of cortical fluctuations (McIntosh et al., 2010) in endogenous resting state EEG (Miskovic et al., 2016) by including a large EEG sample with comprehensive analysis of MSE that includes full high density montage and cluster permutation testing with TFCE alongside developmental changes in PSD.

The maturation of functional brain networks is characterized by the combination of deterministic and stochastic states (Buzsaki, 2006; Sporns, 2011) that are required for complex information processing and adaptation. As an emergent property of cortical development, age-dependent changes in network dynamic gives rise to a level of neural complexity that supports the growing repertoire of behaviours. Increased variability in cortical dynamics is likely a consequence of enhanced functional segregation and integration that occurs between hierarchically organized networks (Luna and Sweeney, 2004) and the enhanced edification of cognitive and behavioural capacity. Some evidence supports this notion and demonstrates that increased EEG signal complexity is associated with functional connectivity (McDonough and Nashiro, 2014; Misic et al., 2011), age-dependent refinements in behavioural control (Garrett et al., 2013; McIntosh et al., 2008), network efficiencies (Misic et al., 2011), and changes from decreased local to increase distributed network variability (Vakorin et al., 2011).

Adolescence is a unique developmental stage during which widespread changes occur across multiple neural, behavioural, and social domains. At the neural level, the transition between childhood and young adulthood is marked by decreases in gray matter, increases in white matter volume, and a shift from local short-range connections to the strengthening of distributed long-range connections (Casey, 2015; Dosenbach et al., 2010; Hwang et al., 2013; Sporns, 2013). With the maturation of long-range connections that support integrated and distributed processing comes neural and cognitive specialization (Beatriz Luna et al., 2020). A hallmark of this specialization during adolescence is the capacity for flexible control over behaviour, which is perhaps most obvious when the demands on self-regulation co-occur with heightened arousal, emotional saliency, and social pressures. The large maturational changes between frontal cortical and subcortical limbic networks are thought to support the development of this “emotional maturity” that allows individuals to appropriately monitor and regulate endogenous states so that they are in line with environmental demands (Johnson et al., 2009; Luna, 2009; Luna et al., 2020). The asynchrony between the maturation of cortical and subcortical networks is thought to be a key factor (Casey, 2015) leading to poorer self-regulation and maladaptive emotional decision-making that is prevalent during the adolescent transition period. For some individuals, these changes in neural architecture and function are coupled with increased vulnerability to psychopathology and poorer self-regulation. Given that the onset of several forms of psychopathology, such as depression and anxiety, coincides with the adolescent period, features of global network dynamics may be informative in the context of other indicators of cognitive ability and mental health.

Recently it has been proposed that the relative shifts in hierarchical (re)organization of connections between and within subcortical and cortical regions across development impact self-regulation capacities, particularly in the context of heightened emotional arousal (Spear and Silveri, 2016). The embedded patterns in neural time series are associated with connectome features (Grady and Garrett, 2018; Mišić, Betzel et al., 2016; Pedersen et al., 2017) and show fair to excellent stability within individuals (Kuntzelman et al., 2018), making them a potentially useful indicator of developmental risk and pathology. Some research has shown that reduced signal complexity is associated with neurodevelopmental disorders and psychiatric outcomes, such as autism, schizophrenia, mood disorders, and Alzheimer’s (Bosl et al., 2011, 2018; Catarino et al., 2011; Escudero et al., 2006; Mišić, Dunkley et al., 2016; Yang et al., 2015). Although resting state activity reflects endogenous rhythms of underlying brain networks and general brain states, there is some evidence to suggest that resting state networks are also systematically engaged when performing event-related tasks (Barnes et al., 2009; Rehme and Grefkes, 2013; Schultz et al., 2012; Smith et al., 2009). Large cohort developmental studies will be important for establishing normative trajectories of dynamic complexity, how deviations confer neurodevelopmental risk, and whether the links between variability and neuropsychological capacity can be exploited to enhance resiliency and self-regulation.

## 5 Limitations, conclusions, future directions

We observed age-dependent increases in brain signal complexity from late childhood into mid adolescence at increasingly longer time scales. In addition, the spatial pattern of results show that increased complexity was most prominent over fronto-central regions, suggesting that these functional changes reflect well known maturation trajectories in brain structure and function into adolescence. These developmental patterns of EEG signal complexity were not captured by age-related shifts in PSD scaling. Although our results align with and extend previous findings from other large studies on brain signal complexity in childhood (Miskovic et al., 2016), the current sample is cross sectional and only includes individuals aged 9–16 years. An absence of longitudinal data, or an adult cohort to examine whether signal complexity continues to change into adulthood, limits a more comprehensive assessment of maturational changes as they relate to EEG complexity. In addition, integrating measures of variability, irregularity, and spectral power will contribute to a deeper understanding of how EEG complexity at multiple temporal scales changes across development (Courtiol et al., 2016; Kosciessa et al., 2020; Polizzotto et al., 2016). Future studies that longitudinally assess intra-individual complexity in resting and task-based EEG, coupled with comprehensive behavioural and neurocognitive assessment, will be especially valuable to our understanding about the functional significance of developmental changes in brain signal complexity.

## Data statement

The data collected as part of this study are currently unavailable.

## Declaration of Competing Interest

The authors declare that they have no known competing financial interests or personal relationships that could have appeared to influence the work reported in this paper.
