# Supplementary material for: Cortical maturation from childhood to adolescence is reflected in resting state EEG signal complexity
Source: Dev Cogn Neurosci. 2021 Mar 23;48:100945. doi: 10.1016/j.dcn.2021.100945 (PMC8027532; doi:10.1016/j.dcn.2021.100945)
Supplement: Supplementary file 1 [file mmc1.docx]

**Supplementary Figure 1**


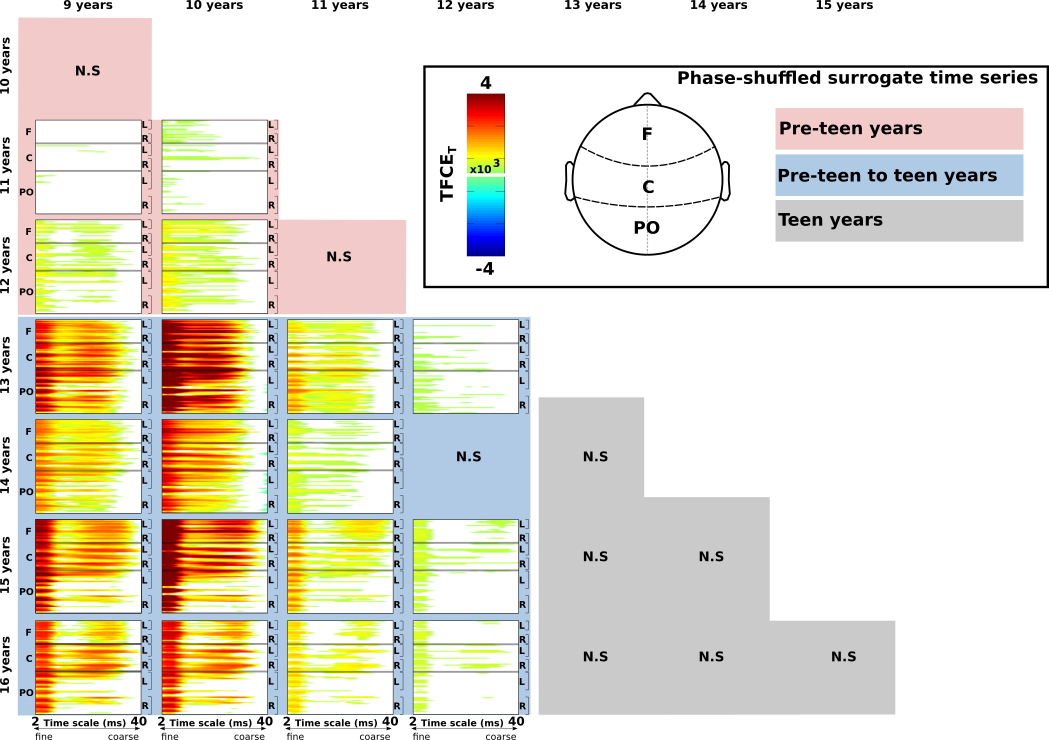


Supplementary Figure 1. Pairwise age group contrasts for each scalp electrode and time scale factor using phase-shuffled surrogate data. Hot colors indicate greater MSE in the older group of a given contrast (e.g., 9 vs 13), masked by significance using Threshold Free Cluster Enhancement with 2000 random between-subjects permutations. No robust effects were observed for decreases in MSE with age. Shaded backgrounds for age group contrasts within pre-teen years (pink), between pre-teen and teen years (blue), and within teen years (grey) . Note: F – frontal; C – central; PO – parieto-occipital. L – left hemisphere; R – right hemisphere. N.S. – no significant contrasts.
